# Supplementary figures and images for: The Role of Vibration Amplitude in the Escape-Hatching Response of Red-Eyed Treefrog Embryos
Source: Integr Org Biol. 2025 Apr 2;7(1):obaf012. doi: 10.1093/iob/obaf012 (PMC11986817; doi:10.1093/iob/obaf012)

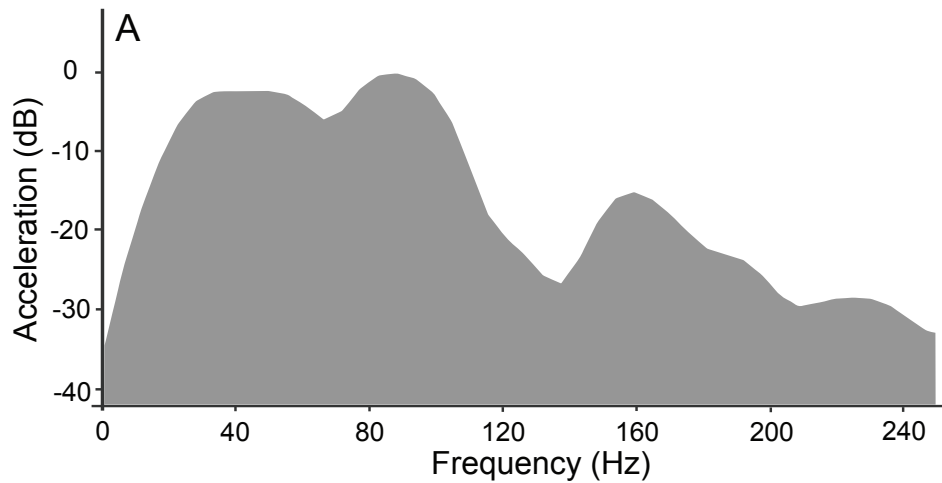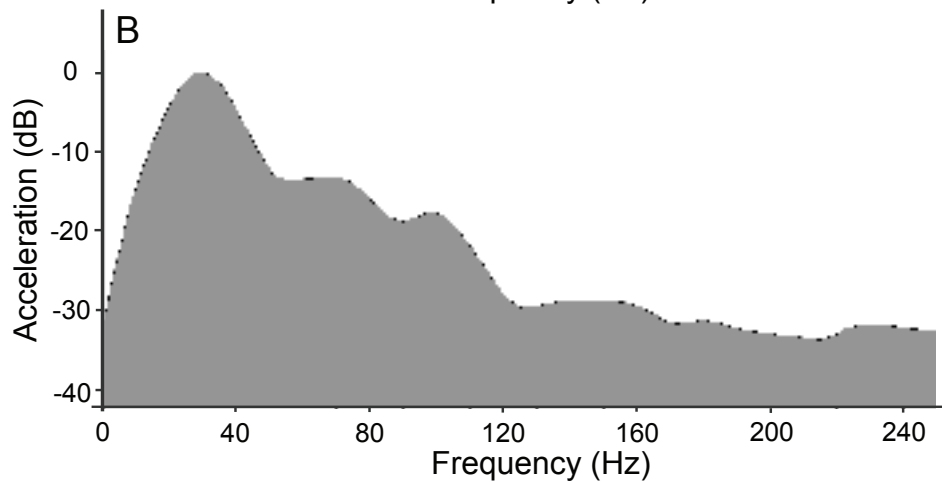

Supplement: obaf012_Supplemental_Files [file obaf012_supplemental_files.zip › FigS1.pdf]

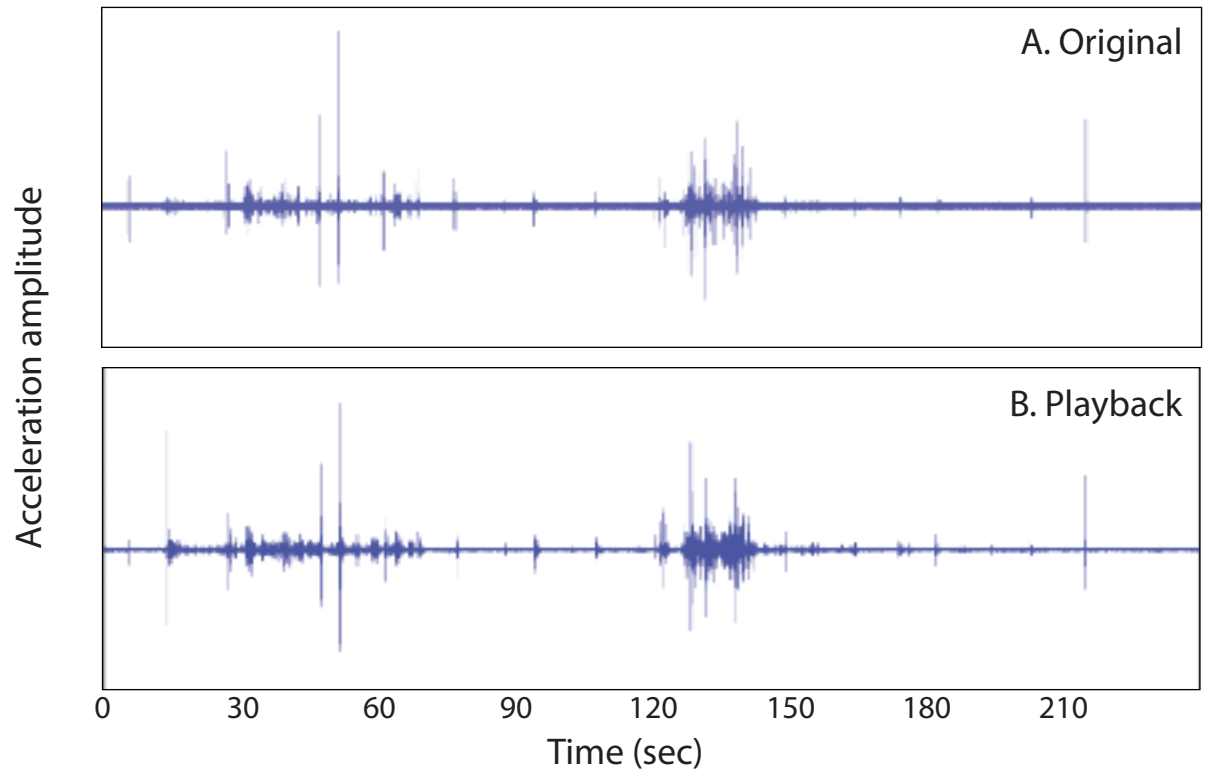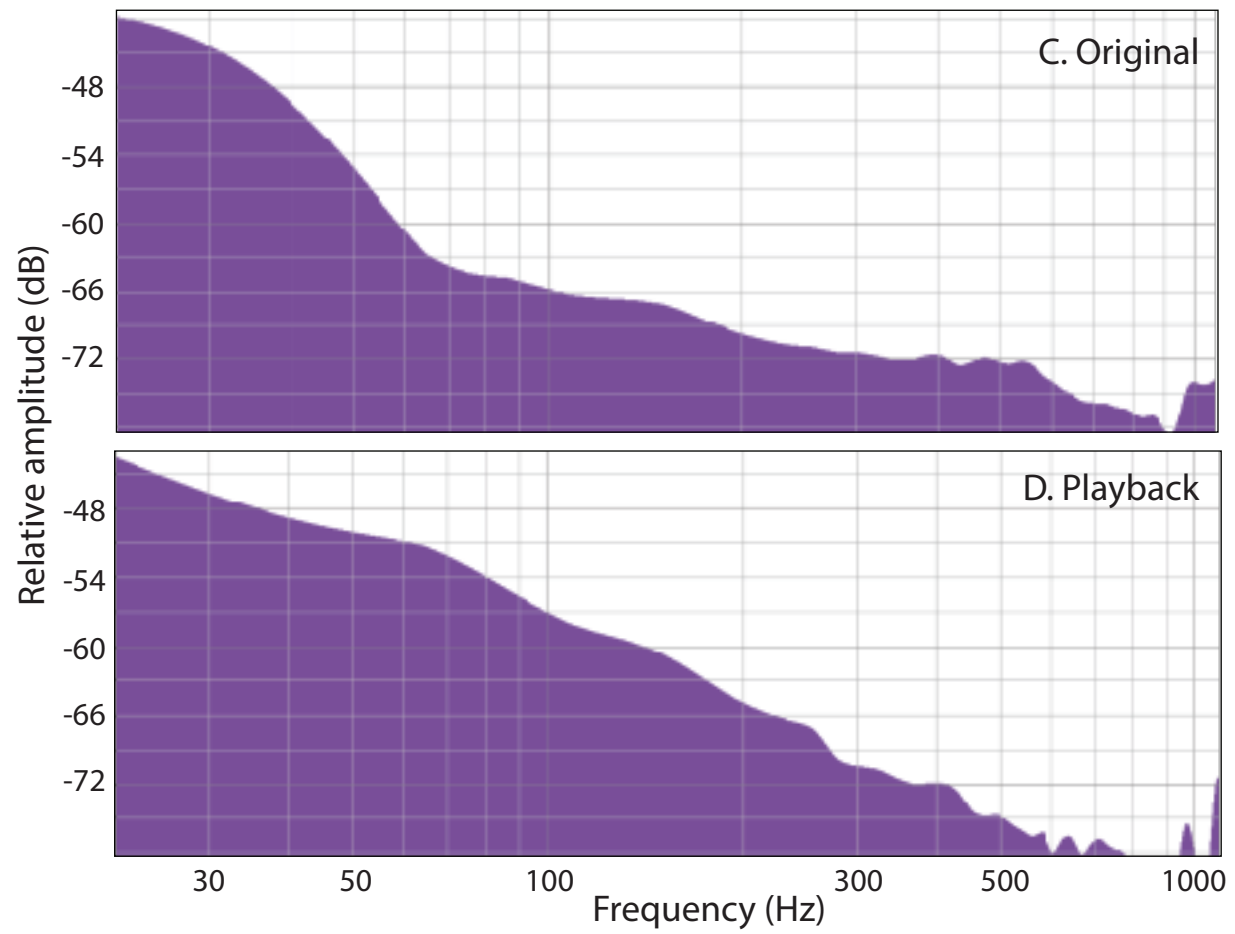

Supplement: obaf012_Supplemental_Files [file obaf012_supplemental_files.zip › FigS2.pdf]

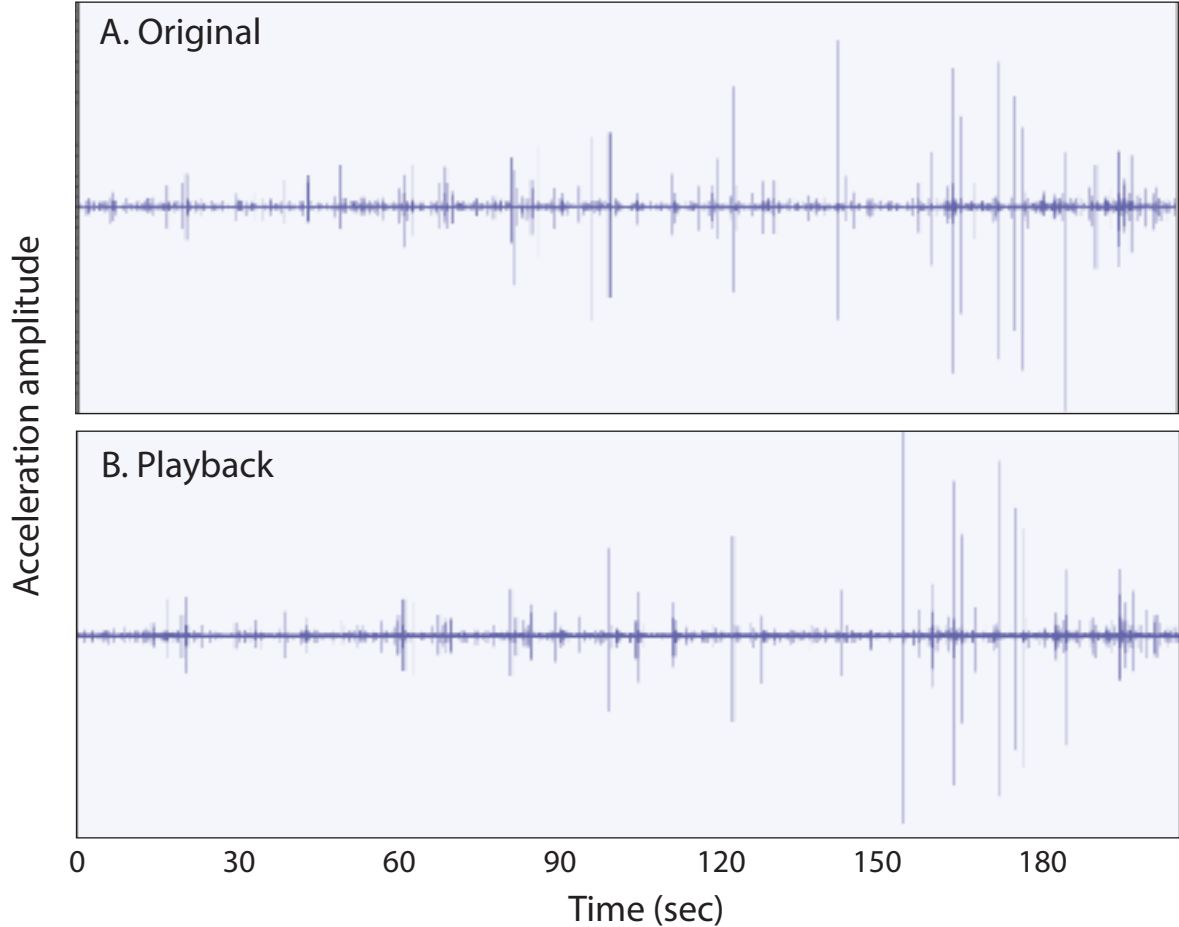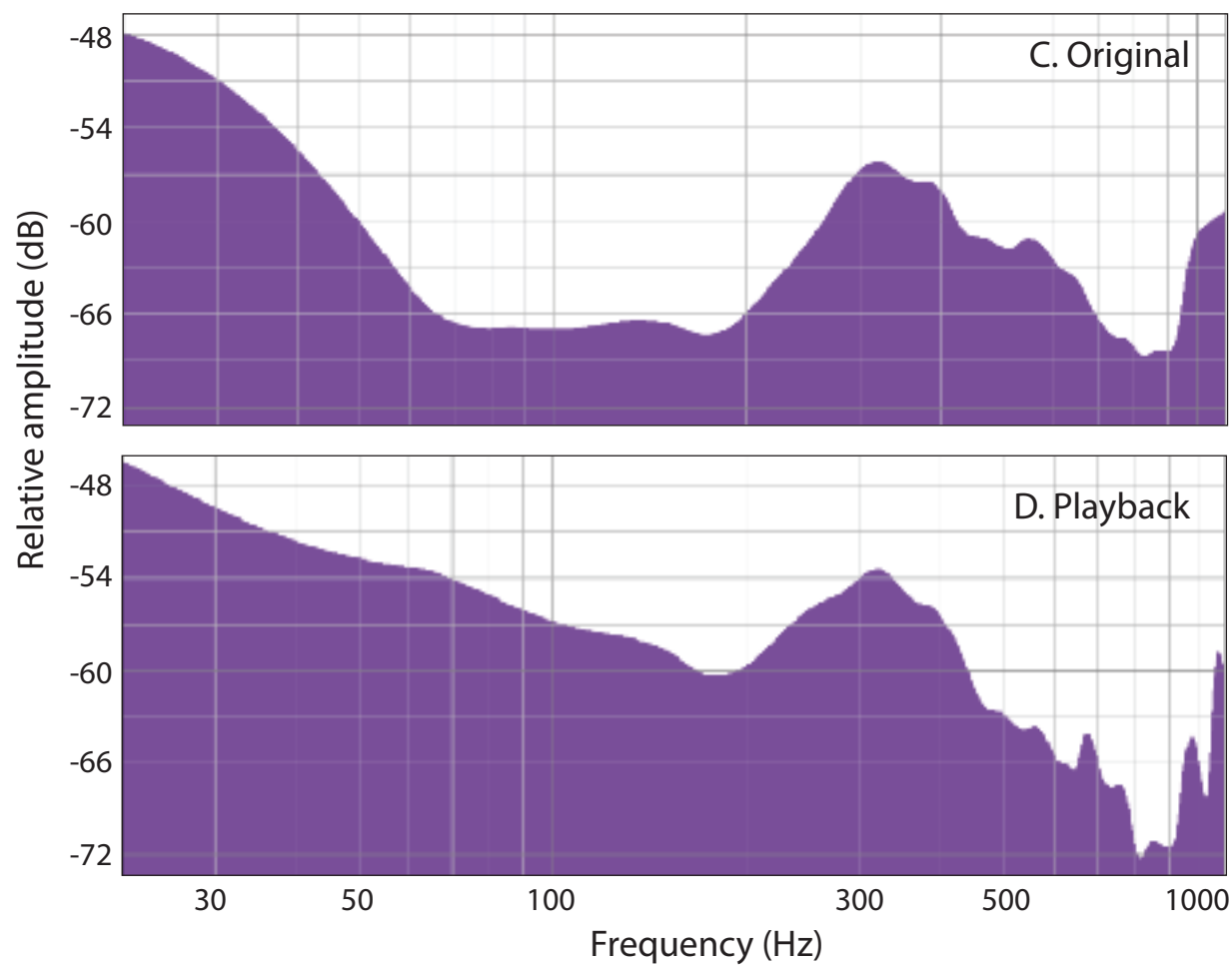

Supplement: obaf012_Supplemental_Files [file obaf012_supplemental_files.zip › FigS3.pdf]
